# CME ACTIVITY: Multicenter GeoSentinel Analysis of Rickettsial Diseases in International Travelers, 1996–2008

**DOI:** 10.3201/eid1511.090676

**Published:** 2009-11

MedscapeCME is pleased to provide online continuing medical education (CME) for this journal article, allowing clinicians the opportunity to earn CME credit. This activity has been planned and implemented in accordance with the Essential Areas and policies of the Accreditation Council for Continuing Medical Education through the joint sponsorship of MedscapeCME and Emerging Infectious Diseases. MedscapeCME is accredited by the Accreditation Council for Continuing Medical Education (ACCME) to provide continuing medical education for physicians. MedscapeCME designates this educational activity for a maximum of 0.75 *AMA PRA Category 1 Credits*™. Physicians should only claim credit commensurate with the extent of their participation in the activity. All other clinicians completing this activity will be issued a certificate of participation. To participate in this journal CME activity: (1) review the learning objectives and author disclosures; (2) study the education content; (3) take the post-test and/or complete the evaluation at **http://www.medscape.com/cme/eid**; (4) view/print certificate.

## Learning Objectives

Upon completion of this activity, participants will be able to:

- Describe the most common rickettsial diseases in returning international travelers between 1996 and 2008.
- Identify risk factors associated with higher likelihood of rickettsial disease among returning international travelers.
- Describe the most common treatment for rickettsial diseases.

## Editor

**Nancy Farm Männikkö, PhD**, Technical Writer-Editor, *Emerging Infectious Diseases. Disclosure: Nancy Farm Männikkö, PhD, has disclosed no relevant financial relationships.*

## CME AUTHOR

**Désirée Lie, MD, MSEd,** Clinical Professor, Family Medicine, University of California, Orange; Director, Division of Faculty Development, UCI Medical Center, Orange, California. *Disclosure: Désirée Lie, MD, MSEd, has disclosed no relevant financial relationships.*

## AUTHORS

Disclosures: ***Mogens Jensenius, MD, PhD; Xiaohong Davis, PhD; Frank von Sonnenburg, MD; Eli Schwartz, MD; Jay S. Keystone, MD, MSc, FRCPC; Karin Leder, FRACP,; Rogelio Lopéz-Véléz, MD, PhD; Jakob P. Cramer, MD; Lin Chen, MD;*** *and* ***Philippe Parola, MD,*** *have disclosed no relevant financial relationships.* ***Eric Caumes, MD,*** *has disclosed the following relevant financial relationships: served an as advisor or consultant for Novartis Pharmaceuticals Corporation; served as a speaker or a member of a speakers bureau for Wyeth France.*

## Earning CME Credit

To obtain credit, you should first read the journal article. After reading the article, you should be able to answer the following, related, multiple-choice questions. To complete the questions and earn continuing medical education (CME) credit, please go to **http://www.medscape.com/cme/eid**. Credit cannot be obtained for tests completed on paper, although you may use the worksheet below to keep a record of your answers. You must be a registered user on Medscape.com. If you are not registered on Medscape.com, please click on the New Users: Free Registration link on the left hand side of the website to register. Only one answer is correct for each question. Once you successfully answer all post-test questions you will be able to view and/or print your certificate. For questions regarding the content of this activity, contact the accredited provider, CME@medscape.net. For technical assistance, contact CME@webmd.net. American Medical Association’s Physician’s Recognition Award (AMA PRA) credits are accepted in the US as evidence of participation in CME activities. For further information on this award, please refer to http://www.ama-assn.org/ama/pub/category/2922.html. The AMA has determined that physicians not licensed in the US who participate in this CME activity are eligible for *AMA PRA Category 1 Credits*™. Through agreements that the AMA has made with agencies in some countries, AMA PRA credit is acceptable as evidence of participation in CME activities. If you are not licensed in the US and want to obtain an AMA PRA CME credit, please complete the questions online, print the certificate and present it to your national medical association.

### Article Title: Multicenter GeoSentinel Analysis of Rickettsial Diseases in International Travelers, 1996–2008

## CME Questions

1. Which of the following are no longer classified as rickettsial disorders?
  A. *Ehrlichia* and *Anaplasma*
  B. *Orientia* and *Coxiella burnetti*
  C. *Coxiella burnetti* and *Bartonella*
  D. *Anaplasma* and *Bartonella*
2. A 44-year-old male traveler returning from Tanzania presents 7 days after return with fever and respiratory symptoms. Among rickettsial diseases to be considered, which of the following is most likely to be the cause of his illness?
  A. Ehrlichiosis
  B. Spotted fever group rickettsiosis
  C. Bartonellosis
  D. Typhus group rickettsiosis
3. Which of the following is least likely to be positively and independently associated with spotted fever group rickettsiosis in a returning international traveler?
  A. Travel for business
  B. Visit to southern Africa
  C. Male gender
  D. Travel from March to May
4. Which of the following is the most commonly used treatment for rickettsial disease among returning international travelers?
  A. Tetracycline
  B. Minocycline
  C. Septra
  D. Doxycycline

### Activity Evaluation

**Table:** 

**1. The activity supported the learning objectives.**				
Strongly Disagree				Strongly Agree
1	2	3	4	5
**2. The material was organized clearly for learning to occur.**				
Strongly Disagree				Strongly Agree
1	2	3	4	5
**3. The content learned from this activity will impact my practice.**				
Strongly Disagree				Strongly Agree
1	2	3	4	5
**4. The activity was presented objectively and free of commercial bias.**				
Strongly Disagree				Strongly Agree
1	2	3	4	5

